# Computational Analysis of Mutations in the Receptor-Binding Domain of SARS-CoV-2 Spike and Their Effects on Antibody Binding

**DOI:** 10.3390/v14020295

**Published:** 2022-01-30

**Authors:** Marine E. Bozdaganyan, Konstantin V. Shaitan, Mikhail P. Kirpichnikov, Olga S. Sokolova, Philipp S. Orekhov

**Affiliations:** 1 Faculty of Biology, Lomonosov Moscow State University, 119991 Moscow, Russia; bozdaganyan@mail.bio.msu.ru (M.E.B.); k.v.shaitan@molsim.org (K.V.S.); kirpichnikov@inbox.ru (M.P.K.); 2 N.N. Semenov Federal Research Center for Chemical Physics, Russian Academy of Sciences, 119991 Moscow, Russia; 3 Faculty of Biology, Shenzhen MSU-BIT University, Shenzhen 518172, China; 4 Institute of Personalized Medicine, Sechenov University, 119146 Moscow, Russia

**Keywords:** coronaviruses, virus–host interactions, binding free energy, antigenic escape, computational mutagenesis, hACE2, SARS-CoV-2, RBD

## Abstract

Currently, SARS-CoV-2 causing coronavirus disease 2019 (COVID-19) is responsible for one of the most deleterious pandemics of our time. The interaction between the ACE2 receptors at the surface of human cells and the viral Spike (S) protein triggers the infection, making the receptor-binding domain (RBD) of the SARS-CoV-2 S-protein a focal target for the neutralizing antibodies (Abs). Despite the recent progress in the development and deployment of vaccines, the emergence of novel variants of SARS-CoV-2 insensitive to Abs produced in response to the vaccine administration and/or monoclonal ones represent a potential danger. Here, we analyzed the diversity of neutralizing Ab epitopes and assessed the possible effects of single and multiple mutations in the RBD of SARS-CoV-2 S-protein on its binding affinity to various antibodies and the human ACE2 receptor using bioinformatics approaches. The RBD-Ab complexes with experimentally resolved structures were grouped into four clusters with distinct features at sequence and structure level. The performed computational analysis indicates that while single amino acid replacements in RBD may only cause partial impairment of the Abs binding, moreover, limited to specific epitopes, the variants of SARS-CoV-2 with multiple mutations, including some which were already detected in the population, may potentially result in a much broader antigenic escape. Further analysis of the existing RBD variants pointed to the trade-off between ACE2 binding and antigenic escape as a key limiting factor for the emergence of novel SAR-CoV-2 strains, as the naturally occurring mutations in RBD tend to reduce its binding affinity to Abs but not to ACE2. The results provide guidelines for further experimental studies aiming to identify high-risk RBD mutations that allow for an antigenic escape.

## 1. Introduction

The recent launch of vaccination campaigns in many countries allowed by the rapid development of several effective vaccines [1] gives hope for a forthcoming amelioration of the world pandemic of SARS-CoV-2. Vaccination will remain the main measure for antiviral protection against COVID19 for a long time, since the development of other types of antiviral drugs is much more time consuming [2].

In [3], it was firstly shown that ACE2 acted as the receptor for SARS-CoV, later in 2020 in [4], researchers provided one of two early experimental structures of the SARS-CoV-2 RBD–ACE2 complex, showing how the Spike protein recognizes its receptor. In [5,6], it was demonstrated that the Spike protein of SARS-CoV-2 and especially its receptor-binding domain (RBD) is one of the major targets of neutralizing antibodies elicited by natural infection or vaccination [7]. The titers of IgM and IgG antibodies against the receptor-binding domain (RBD) of the Spike protein of SARS-CoV-2 decrease significantly over 6 months, with IgA being less affected [8]. At the same time, a number of recent studies have identified viral mutations that escape neutralizing antibodies targeting the SARS-CoV-2 Spike protein [9,10]. Some of these mutations are already present in the human population [11], but many more may be present in natural reservoirs of coronaviruses and represent a potential threat [12,13]. These observations raise concerns about the potency of monoclonal antibodies as well as the protective efficacy of the existing vaccines [14].

Major efforts have been undertaken by the scientific community in order to classify existing data with bioinformatic resources [15,16,17,18] and map potentially hazardous mutations [19]. Particularly, several sites at the SARS-CoV-2 Spike protein, which reduce the neutralizing activity of monoclonal antibodies, and/or their cocktails/human sera, were identified, including E484K, K417N [11], N439K [20], E406W [19], N501Y [21], and others [10]. Many of these mutations occur in the receptor-binding domain (RBD) of Spike, which mediates binding to the angiotensin-converting enzyme 2 (ACE2) receptor, resulting in the virus entry into the cells. As mentioned above, the majority of leading anti–SARS-CoV-2 antibodies also target this domain [6,22,23] rendering these mutations especially risky.

Due to the central role of RBD domain as a key Ab target, in the present study, we carried out a comprehensive computational investigation of the effects which may be induced by its mutations on the affinity to various neutralizing antibodies and hACE2 exploiting structural data available to date. We classified RBD-targeting antibodies and revealed that their epitopes demonstrate remarkable variance in predicted binding energies, the degree of occlusion by the glycan chains, and conservativity. We further assessed the impact of naturally occurring residue replacements in RBD to possible antibody resistance and the ACE2 binding. Furthermore, analyzed the potential outcomes of all possible RBD mutations. We believe that the thorough virtual mutagenesis analysis reported here will guide further experimental studies of Ab resistance and identification of natural SARS-CoV-2 variants capable of antigenic escape.

## 2. Materials and Methods

### 2.1. Analysis of Atomic Structures and Clustering

The structures of all RBD-Ab complexes were retrieved from the PDB database (see Table 1). In order to classify Ab epitopes, each Ab-RBD interface was encoded as a binary vector with the length equal to the number of residues in the reference RBD structure (SARS-CoV-2 Spike protein in complex with the ACE2 receptor, the PDB code 6M17). Positions corresponding to residues in contact (distance between any pair of heavy atoms less than 6 Å) with an Ab were set to 1, while those not forming contacts were set to 0. The encoded epitopes were further clustered by means of the hierarchical clustering algorithm and split into four clusters based on the inspection of the inter-cluster distances. The PDB structure 6M17 of the Spike protein in complex with the ACE2 receptor resolved by Cryo-EM to 2.90 Å [24] was used to estimate the effects of mutations on the RBD binding to ACE2.

### 2.2. Estimation of Binding Energies

For the calculation of binding energies between Abs, ACE2 and RBD, we used PRODIGY [42,43]. The contributions of amino acid mutations to the binding energies were estimated using the BeAtMuSiC server based on application of a statistical model to coarse-grained models of protein–protein complexes [44].

Briefly, the approach predicts a change in protein–protein binding affinity upon a mutation by combining the output of two binding models, one considering that both partners of the interaction are able to fold independently of each other with the change of affinity given by:

(1) $$\Delta \Delta G_{B^{\prime }}=\Delta \Delta G_{C}-\left(\Delta \Delta G_{P1}+\Delta \Delta G_{P2}\right),$$

and another assuming that the partners are unable to fold independently, and the change in binding-free energy on mutation is given just by:

(2) $$\Delta \Delta G_{B^{\prime \prime }}=\Delta \Delta G_{C},$$

where ΔΔ*G_P_*_1_ and ΔΔ*G_P_*_2_ are the respective folding free energies of the two partners, and ΔΔ*G_C_* is the folding free energy of the protein–protein complex.

In turn, the effects of mutations on folding free energies can be found using the following expression:

(3) $$\Delta \Delta G=\sum_{i=1}^{13}\alpha_{i}\left(A\right)\Delta \Delta W_{i}+\alpha_{14}\left(A\right)\Delta V_{+}+\alpha_{15}\left(A\right)\Delta V_{-}+\alpha_{16}\left(A\right),$$

where the first term is a sum of energetic changes, $\Delta \Delta W_{i}$, induced by the mutation according to a set of statistical potentials extracted from a data set of known protein structures and describing the correlations between amino acid types, pairwise inter-residue distances, backbone torsion angles and solvent accessibilities. The terms $\Delta V_{+}$ and $\Delta V_{-}$ account for the possible creation of packing defects and depend on the change of the side chain volume. The weights $\alpha_{i}$ are sigmoid functions of the solvent accessibility A of the mutated residue, and they were fit on the basis of a data set of experimentally measured changes in folding free energy.

We gave preference to the BeAtMuSiC approach showing satisfactory results [45], rather than potentially more accurate methods based on molecular dynamics calculations, in order to accelerate analysis and to be able to extend it to a large set of all possible RBD variants, which would otherwise become computationally infeasible. The effects of multiple mutations were estimated as the sum of individual contributions. Although multiple mutations may have a synergistic effect on binding, we took this approximation for the sake of speeding up the calculations.

### 2.3. Sequence Analysis

The sequences of the variants of the SARS-CoV-2 Spike protein were obtained from a database supported by the GISAID initiative [46] accessed on 15 November 2021. This database served as a source of information about novel RBD variants and the dates of their emergence. A multiple sequence alignment (MSA) was obtained using MAFFT version 7 [47] using the default settings and truncated to the region corresponding to the RBD (C336-L518) present in the reference structure (PDB code 6M17) for further analysis. The identical RBD sequences were sorted, resulting in 3370 unique sequences and the variability of MSA positions was estimated in terms of Shannon entropy using ProDy [48]:

(4) $$H=-\sum_{i=1}^{M}P_{i}log_{2}P_{i},$$

where $P_{i}$ is a probability of *i*-th amino acid at the given position in MSA. The lower and upper limits of Shannon entropy correspond to fully conserved and fully random (equal probability of all twenty amino acid types) amino acids at the given position.

In turn, the interface variability was estimated as the average entropy of amino acid positions forming the interface.

Mutations in variable positions along the protein chain can occur because they are either accompanied or preceded by compensatory changes in other variable positions. Such compensation would result in a coupling between changes in the two positions, or coevolution. We used Mutual Information (MI) to identify such potentially co-evolving (i.e., dependent on each other) amino acid residues in RBD as implemented in ProDy [48]. MI corresponds to the reduction of uncertainty (as measured by Shannon entropy defined by Equation (4) of random variable X given random variable Y (and vice versa):

(5) MI(X,Y)=H(X)−H(X|Y)=H(Y)−H(Y|X). 

Since the inclusion of related sequences in the MSA may lead to an overestimation of the conservation and coevolution propensities by including those amino acids that retain their identity due to common ancestry, we applied a correction the MI matrix as suggested in [49].

The positions were separated by less than 20 Å in the reference RBD structure (6M17) and those regarded to be in a direct contact were set to zero in the MI matrix in order to remove such direct effects from consideration.

### 2.4. Analysis of Epitope Accessibility in the Glycolisalted S Protein

To assess the alterations in epitope accessibility induced by the presence of post-translational modifications (PTMs), we calculated the relative change of solvent-accessible surface area (SASA) for each residue in RBD in the glycosylated model of Spike protein and in the absence of PTMs. The analysis was performed for a molecular dynamics trajectory of the fully glycosylated Spike protein trimer in a viral membrane, based on the experimental structure of 6VSB reported in [50] and freely available at the CHARMM-GUI platform [51] (system 6VSB_1_1_1). SASA was estimated using the standard Gromacs [52] tool, *gmx sasa*. The resulting value corresponds to the relative decrease of a residue SASA in the presence of glycan chains averaged over MD trajectory and 3 individual monomers.

## 3. Results and Discussion

### 3.1. Analysis of Epitopes Reveals Distinct Clusters of Ab Binding Poses with Unique Features

**Figure 1:**
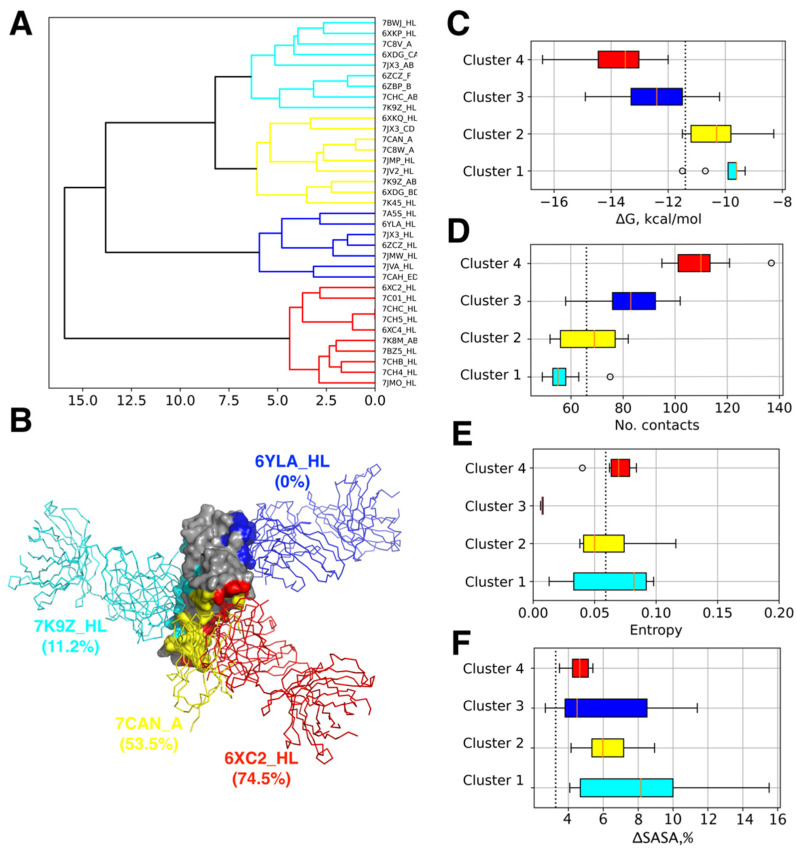
Cluster analysis of antibody epitopes. (**A**) Hierarchical clustering dendrogram of binding epitopes. Four major clusters of epitopes are colored in cyan, yellow, blue, and red. (**B**) Structures of representative antibody-RBD complexes for each cluster captioned with its PDB code. Colors are corresponding to panel A. Epitope overlaps with the ACE2 binding site are given in the parenthesis. (**C**) Binding free energies; (**D**) number of contacts between Ab and RBD; (**E**) interface variability in terms of average entropy; (**F**) mean per-residue change of solvent accessible surface area (SASA) in the presence of glycans with respect to the unglycosylated S-protein for epitopes of each cluster of analyzed Ab-RBD complexes. Corresponding values for the ACE2-RBD complex are shown by the dotted line.

The majority of human neutralizing antibodies (Ab) against SARS-CoV2 S-protein bind to diverse epitopes at the surface of its receptor-binding domain (RBD) preventing its attachment to the host cell and thus neutralizing the virus [53]. RBD accounts for 90% of serum neutralizing activity [31]. In order to explore the plasticity of their binding modes and classify them on the basis of residues involved in the complex formation with RBD, we applied hierarchical clustering to the binding interfaces as described in the Methods. The analysis was performed for 35 of the Ab-RBD complexes available in the PDB database (Table 1). We further considered four major clusters of epitopes based on the distances observed in the clustering dendrogram (Figure 1A). Three of these clusters approximately correspond to three groups of RBD-targeting Abs suggested before [7]. However, our analysis revealed an additional distinct group of epitopes, cluster 3, including Abs such as synthetic nanobody MR17-K99Y and monoclonal Ab S2H14 (see Table 1).

A representative complex for each cluster was chosen based on the estimated binding-free energy. In each cluster, the Ab-RBD complex with the lowest binding free energy was selected for further analysis (hereafter referred by their names or PDB codes with the prefix standing for the Ab chains in the corresponding structure: 52 Ab/7K9Z_HL (cluster 1, cyan), MR17-K99Y/7CAN_A (cluster 2, yellow), CR3022/6YLA_HL (cluster 3, blue), and CC12.1/6XC2_HL (cluster 4, red)). It may also be noted that complexes with lower binding energy Abs tend to form more contacts to RBD (see Figure S1) implying that focusing our analysis specifically on them should also allow us to examine the corresponding clusters of epitopes in a more complete way in addition to being concentrated at potentially the most affine Abs.

Detailed inspection of the interfaces in each cluster (Figure 1B) suggests that three extend over the same region of RBD encompassing the receptor-binding motif (RBM) β-hairpin of the RBD and partially overlap with each other and with the ACE2 binding site (clusters 1, 2, and 4; the average overlap with the ACE2 site is 11.2, 53.5, and 74.5%, respectively). However, the fourth cluster (cluster 3) occupies a distinct area on the RBD surface including residues 368-388 forming two α-helices and an intervening β-strand. This cryptic epitope is remote from the ACE2 site and not overlapping with it or epitopes of other clusters of Abs at all. While the Abs binding to this site do not prevent the RBD interaction with ACE2 in the direct way they sterically clash with ACE2 interacting with the same protomer within an S trimer [31].

**Figure 2:**
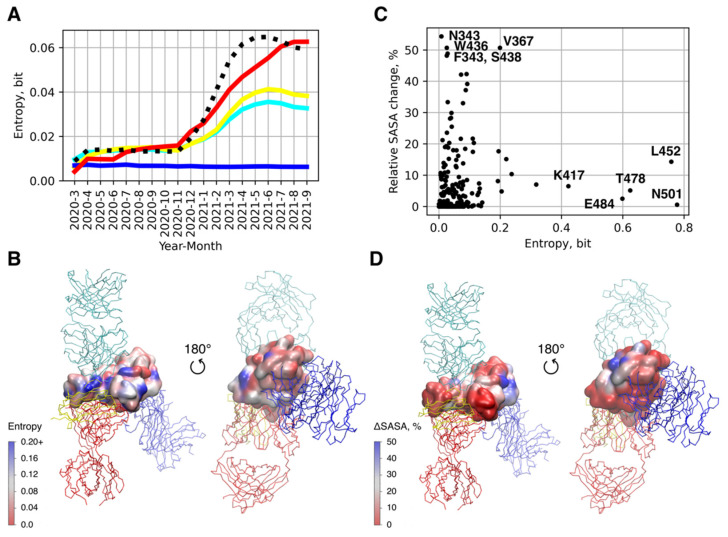
Analysis of conservativity and epitope accessibility in RBD of SARS-CoV-2 Spike. (**A**) Mean Shannon entropy of amino acid positions belonging to Ab/ACE2 interfaces in multiple sequence alignment (MSA) of RBD sequences deposited to a given date as a measure of the interface variability. (**B**) Shannon entropy of individual amino acid residues of RBD mapped onto its surface. The positions of the four investigated antibodies are shown with respect to RBD. (**C**) Per-residue change of solvent accessible surface area (unglycosylated/glycosylated S protein) plotted against the entropy of corresponding position in MSA; (**D**) Per-residue change of solvent accessible surface area (unglycosylated/glycosylated S protein) mapped onto the RBD surface.

Interestingly, the identified groups of Abs appear distinct in terms of their predicted binding free energy and number of protein–protein contacts. While Abs belonging to clusters 1 and 2 form a similar or smaller number of contacts to RBD compared to ACE2, and they have comparable or lower predicted affinity (Figure 1C,D), the Abs of clusters 3 and 4 have larger numbers of contacts resulting in lower binding free energies even exceeding the corresponding values for ACE2. The analysis of epitope shielding by glycans, based on the assessment of solvent-accessible solvent area in the molecular dynamics trajectory of the fully glycosylated Spike trimer [50], indicates that binding interfaces of Abs in cluster 4 and partially in cluster 3 are less occluded by the glycan chains as compared to epitopes belonging to three other clusters (Figure 1F and Figure 2D) suggesting that they can remain more accessible allowing bound Abs to make more contacts to RBD [54].

Further analysis of amino acid variability of the Spike RBD sequences available to date reveals that clusters 1, 2, and 4, which are spatially adjacent and overlap to some degree with the ACE2 interface, exhibit a significant level of sequence variability measured by means of Shannon entropy normalized by a number of amino acids comprising the epitope (see Figure 1E). Their average entropy is comparable with that of the ACE2 interface, suggesting that both the receptor interface and the epitopes of these Abs are present in multiple variants circulating in the population. This also implies a significant evolutionary adaptation undergone by these RBD epitopes due to the selection of variants, which are capable of escaping the immune response and/or enhance virulence [55]. It is also worth mentioning that the most variable amino acid positions in RBD demonstrate low occlusion by the glycans. Contrarily, the positions with the highest degree of relative glycan screening are quite conservative (Figure 2C and Figure S3). Altogether, it implies that the unshielded regions targeted by Abs experience the strongest evolutionary pressure, which, in its turn, results in their higher sequence variability.

In contrast to the Abs of clusters 1,2, and 4, the epitope of Abs belonging to cluster 3, which does not overlap with the ACE2 interface at all, demonstrates very low sequence variability, implying that the majority of Spike sequences lack diversity in this region, which is thus unlikely to experience strong selection. Given quite a high predicted affinity of Abs targeting this epitope (Figure 1C), this observation seems surprising as it implies that these Abs do not significantly contribute to the natural human immune response despite their promising characteristics. One of the possible explanations comes from the structural analysis of CR3022 Ab belonging to this group in complex with the Spike. It shows that this epitope is only accessible when the RBD is in the up conformation in at least 2 Spike proteins within the trimer while the steric clashes with the bound antibodies appear otherwise [56]. Overall, the lack of strong evolutionary pressure on this epitope confirms previously proposed assumption that the group of Abs targeting it are likely immunosubdominant [7].

We also assessed how the variability of different epitopes/ACE2 interfaces has changed during the course of pandemic as well as how the novel missense mutations were accumulated in these regions of RBD. The results clearly show that novel mutations appear in the all analyzed epitopes regardless of their proximity to the ACE2 interface at about the same rate, which, as expected, decreases during the course of pandemics (Figure S2) as the pool of novel unique mutations, which are beneficial or non-deleterious, is limited and rapidly depletes over time.

At the same time, the variability of three out of the four investigated epitopes, i.e., those of 52, MR17-K99Y, and CC12.1 Abs, underwent a significant increase in 2021 compared to its rather low values observed before, which did not noticeably change till the end of 2020 (Figure 2A). Similar trend is observed for the ACE2 interface. Contrary to them, the variability of the CR3022 epitope does not experience any changes during the whole period of pandemics and it remains very low until now suggesting its high conservativity among the all of variants ever spread in the population.

The observed increase in variability of three epitopes overlapping with the ACE2 interface may result from the epistatic effects [57] of some RBD variants [58], which became widespread in the population during late 2020 and allowed for the fixation of otherwise compromised mutations. Intriguingly, this increase coincides in time with the period from ca. June 2020 to January 2021, when the rate at which the novel unique mutations emerged in RBD seems steady in contrast to the exponential decrease in the accumulation rate of novel mutations during both the previous and the consequent months (see Figure S2). One may hypothesize that it reflects the emergence of novel mutations during this time, which were epistatically dependent on previously emergent mutations and which could not be otherwise tolerated. Indeed, the analysis of coevolved (i.e., synchronously mutating) amino acid residues in RBD by means of estimation of mutual information (MI) reveals coupling between spatially remote positions separated by more than 20 Å (see Figure S4, the residue pairs with the highest coupling are N501-E484, N501-T478, N501-K417, E484-K417, and T478-L452). Such coupling may result from the indirect influence of one residue on other positions through some intermediate residues; however, it can also signify allosteric coupling in some cases [59] in line with the idea of possible epistatic interactions between certain positions in RBD. This quite intriguing hypothesis is worth further scrutiny.

Epistasis has previously been studied intensely in the influenza surface protein hemagglutinin, and positive epistasis was detected in several regions of the hemagglutinin receptor-binding domain [60]. In relation to the Spike protein of SARS-CoV-2, epistatic mutations could, for instance, allow the Spike protein to adopt a specific conformation enabled by the presence of a distinct set of mutations resulting in unique protein structural and dynamical features. For instance, a recent study demonstrated the impact of antigenicity and infectivity of the Spike D614G SARS-CoV-2 variant in combination with different other mutations [61]. The study shows that D614G alone only slightly affects the infectivity, but in combination with some mutations in Spike, they can both increase and decrease viral infectivity to a larger extent. Similar findings have been reported regarding potential escape from single-domain antibodies. Similarly, D614G alone cannot escape nanobodies. However, the combination of D614G with other Spike mutations can enable the antibody escape [61]. This data together with our coevolutionary analysis suggests that the emergence of epistatic mutations in Spike protein will likely be involved in further adaptability of SARS-CoV-2, including the enhanced transmissibility, stability, and antibody resistance.

### 3.2. Single RBD Mutations Have Limited Effect on its Binding Affinity to Abs and ACE2

**Figure 3:**
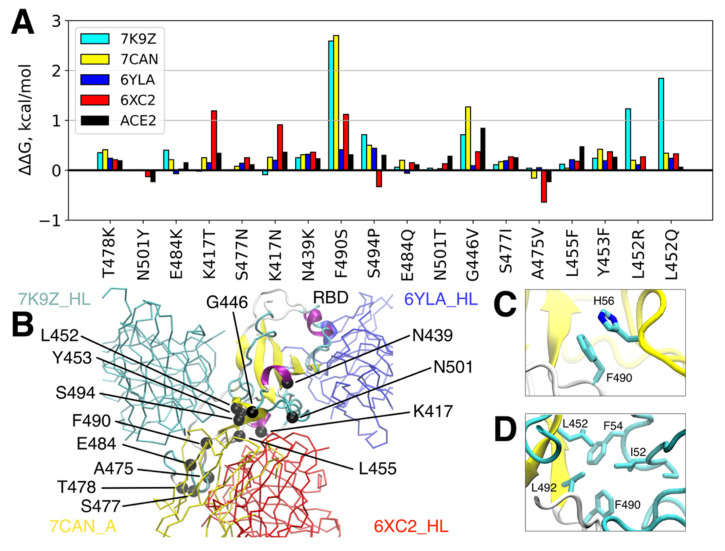
Influence of widely spread individual amino acid replacements on the affinity between antibodies/ACE2 and RBD. (**A**) Predicted alteration of the binding free energy for the 16 most spread in the population RBD mutations and 2 recent mutations of L452 particularly found in δ, δ+, ε, κ, and λ strains; (**B**) Locations of the mutated residues at RBD along with the spatial orientation of the 4 representative antibodies are shown. (**C**) Intermolecular interaction between F490 of RBD and H56 of MR17-K99Y antibody (PDB: 7CAN). (**D**) Intermolecular interaction between F490 and L452 of RBD and their hydrophobic environment comprising I52 and F54 of the 52 antibody (PDB: 7K9Z).

A substantial number of mutations in the RBD can be well tolerated without noticeable consequences for ACE2 binding [62]. This raises the concern of antigenic escape, which may severely limit the therapeutic potential of neutralizing Abs [63]. We employed virtual mutagenesis analysis in order to assess the potential effects of point mutations in RBD on its binding affinity to antibodies belonging to four identified clusters and to the SARS-CoV-2 natural receptor, ACE2. Firstly, we estimated the effects on the binding energy for those single amino acid substitutions that are the most common mutations in the reported viral genomes (Table 2). While almost all of them (except for A475V, which might slightly increase affinity towards Ab CC12.1, see Figure 3) appear to decrease the binding affinity to either all studied Abs (e.g., N439K) or some of them (e.g., E484K), this effect is low. Notable exceptions are the G446V and K417N RBD variants, for which the binding energy is increased by 1.3 kcal/mol and 0.9 kcal/mol towards MR17-K99Y (PDB: 7CAN, cluster 2) and CC12.1 (PDB: 6XC2, cluster 4) Abs, respectively. These ΔΔG values should lead to ~8.2 and ~4.4 fold-change drops in K_D_ of MR17-K99Y (PDB: 7CAN) and CC12.1 (PDB: 6XC2) Abs, respectively, at 310 K assuming the predicted binding-free energies for these Ab-RBD complexes (see Table 1). At the same time, most mutations also result in lower affinity to ACE2. Remarkably, the most widespread RBD mutation in the population to date, N501Y, which is particularly specific to α, β, γ, θ, μ, and ο variants, either does not cause any changes in affinity (to 52, MR17-K99Y, and CR3022 Abs) or even results in a slight increase in the affinity to CC12.1 Ab and the native receptor, ACE2, which might account for the fixation of this mutation in the population. It is also worth mentioning that the affinity of CR3022 (PDB: 6YLA), the representative Ab of cluster 3, seems to be the least affected by the analyzed mutations compared to Abs representing the other three clusters. This is apparently due to the remoteness of its epitope from the most frequent mutations, which mainly occur in the variable RBM region of RBD.

Overall, these results suggest that none of the currently widespread single mutations of RBD are able to completely break the binding between RBD and the broad spectrum of Abs targeting it. However, two mutations may lead to partial loss of affinity to some of the analyzed Abs, such as K417N and G446V. The former replacement, which is specific for the B.1.351 lineage (known as the South African variant) has already been demonstrated to reduce the affinity to sera/monoclonal Ab [64].

Since CoVs undergo continuous and extensive antigenic evolution [65], which can potentially lead to amino acid variations that are not currently observed but can appear in future, we did not limit our analysis to the existing variants but performed the complete virtual mutation scanning of RBD (notably, the least conserved domain of Spike [20]) by systematically changing all amino acid positions to 19 alternatives. The latter task was allowed here by exploiting the fast computational approach for virtual mutagenesis.

**Figure 4:**
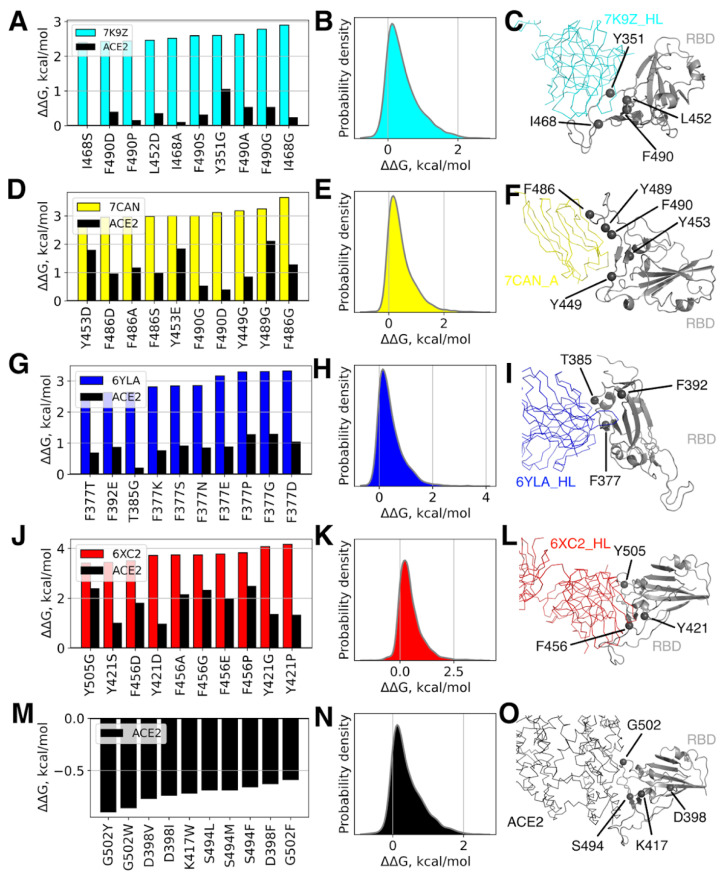
Effects of single mutations on the binding affinity of the RBD-antibody and RBD-ACE2 complexes. RBD mutations with the largest positive (i.e., destabilizing, for different antibodies, (**A**,**D**,**G**,**J**)) and negative (i.e., stabilizing, for ACE2, (**M**)) predicted contributions are shown in the left panels; the distributions of predicted affinity alterations caused by all the possible single mutations are shown in the middle panels (**B**,**E**,**H**,**K**,**N**); amino acid positions with the largest predicted contribution to the binding affinity in the structures of corresponding protein complexes are shown in the right panels (**C**,**F**,**I**,**L**,**O**).

The effects of the majority of mutations on the binding affinity is very low, as indicated by corresponding distributions of the predicted ΔΔG values (Figure 4, middle panel). However, all of these distributions are not symmetrical but have a longer right tail corresponding to positive ΔΔG values and, thus, the decrease in affinity. In other words, the number of mutations that lower affinity and the extent of this lowering effect is greater than for mutations that strengthen the binding. This observation is also true for the RBD-ACE2 interaction (see Figure 4N).

Despite the very low effects induced by the vast majority of mutations on the RBD-Ab/ACE2 binding energy, some mutations may potentially lead to a change by up to +3–4 kcal/mol, which might already reduce the affinity between proteins drastically, given the typical Ab-RBD binding energies (see Table 1, ΔG_average_ = −11.7 kcal/mol; experimental values fall into the same range, e.g., for CR3022 K_D_ = ~6.6 nM–~115 nM [36,56,66] corresponding to ΔG = −11.6–-9.8 kcal/mol; for ACE2–K_D_ = ~15nM [22], i.e., ΔG = −11.1 kcal/mol at 310 K).

**Figure 5:**
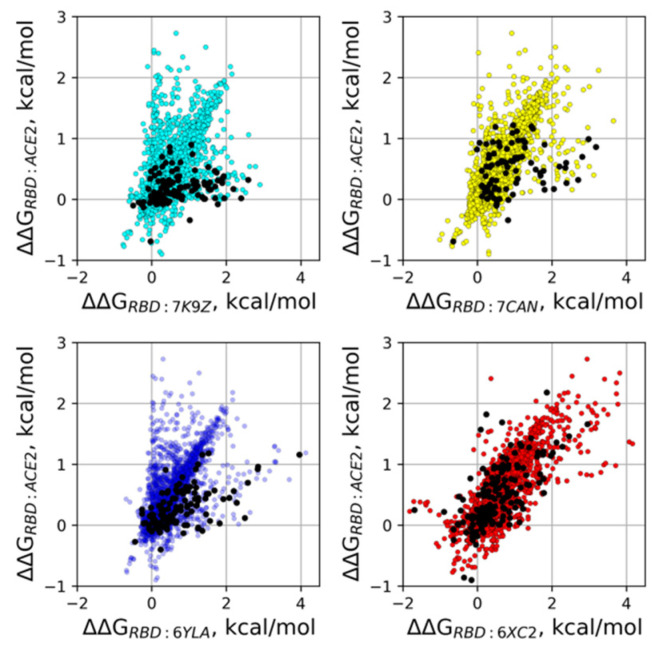
Analysis of single amino acid RBD mutants. Affinity changes of all possible single amino acid RBD mutants to different antibodies plotted against the respective affinity changes to ACE2. The black dots correspond to the mutations observed in real sequences.

Importantly, mutations with strong effects on the binding energy usually occur directly or in a close vicinity of an Ab epitope, often also resulting in the reduced affinity of RBD towards ACE2, since for the majority of investigated Abs their epitopes overlap with the ACE2 interface. Indeed, we could observe a positive correlational trend between ΔΔGs estimated for mutations in the RBD-ACE2 complex and ΔΔGs for the same mutations in all of the studied Ab-RBD complexes (see Figure 5, Pearson’s r = 0.59÷0.81). This fact may account for the lack of a major fraction of such deleterious mutations in real SARS-CoV-2 sequences, due to their interference with the RBD-ACE2 binding. Indeed, the comparison of the effects of real RBD mutations observed in deposited SARS-CoV-2 sequences onto Abs and ACE2 binding implies that they tend to reduce RBD-ACE2 binding to a lesser degree as compared to their effect on RBD-Ab affinities (see black dots in Figure 5). In other words, it appears that the mutations that promote antigenic escape but at the same time do not affect ACE2 binding are preferably selected during the SARS-CoV-2 evolution. Quite surprisingly, this trend is pronounced for 52 (PDB: 7K9Z), MR17-K99Y (PDB: 7CAN), and CR3022 (PDB: 6YLA) Abs but not for CC12.1 (PDB: 6XC2). A possible explanation for this discrepancy might be that the epitope of the latter Ab has the largest overlap with the interface of ACE2, leaving very little room for such adaptations in RBD, which lead to the antigenic escape from these Abs without affecting the ACE2 binding.

### 3.3. Antigenic Escape Assessment for Existing SARS-CoV2 Variants with Multiple Mutations

Finally, we estimated potential changes of the binding energy between RBD and four studied Abs caused by multiple mutations co-occurring in the real viral sequences stored in the GISAID database to date. The total affinity change was estimated as a sum of the binding energy changes induced by all the individual mutations in a given sequence, and compared with the cognate change of the RBD affinity towards ACE2.

**Figure 6:**
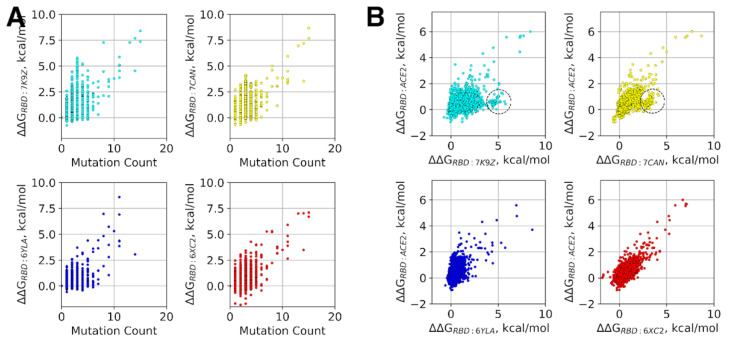
Analysis of RBD variants. (**A**) Affinity changes of RBD variants to different antibodies as a function of the total count of mutations in an RBD mutant; (**B**) Affinity changes of RBD variants to ACE2 are plotted as a function of the respective affinity changes to different antibodies. Dashed cycles indicate RBD variants with significant decrease of affinity towards antibodies, which is not accompanied by any notable change of affinity to ACE2.

In general, the results indicate that a higher number of mutations in RBD expectedly leads to larger ΔΔG (see Figure 6A, Pearson’s r = 0.57 ÷ 0.84). At the same time, as few as two substitutions are already sufficient to reduce the RBD affinity to individual Abs by up to 2.5–3.0 kcal/mol, while six substitutions may result in ΔΔG up to 3.3–4.0 kcal/mol. Some SARS-CoV2 variants, including the variants with the largest estimated change in RBD-Ab/ACE2 affinity and widely spread variants, are listed in Table 3.

None of the widely spread variants of concern except the recently emerged B.1.1.529 (ο) seem to be able to escape the studied Abs efficiently. The RBD of the latter variant carries five times more mutations than any previously concerned variant of SARS-CoV-2 and may indeed be potentially resistant against some groups of Abs, including 52 and CC12.1. On the other hand, this developed resistance likely comes at a cost of reduced affinity to ACE2, as indicated by the corresponding ΔΔG values. Both observations find support in the preliminary experimental reports, thereby indicating both an increased ability of the omicron variant to avoid Abs and its decreased morbidity [67,68].

The most drastic decrease in affinity was predicted for MR17-K99Y (7CAN) and CC12.1 (6XC2) Abs towards the RBD variants found in animals, i.e., bats and pangolins. These RBD sequences comprise as many as 20–25 mutations. At the same time, the largest decrease in the affinity towards 52 (7K9Z) and CR3022 (6YLA) Abs was estimated for the two sequences obtained from human samples in Iran and accommodated 8 and 11 substitutions, respectively. In all of these cases, the predicted ΔΔG (~4.3–~9.5 kcal/mol) may potentially lead to the complete evasion of these variants from specific Abs. It is also worth mentioning that all of the variants with the largest affinity decrease towards particular Ab also, to some extent (at least by ~2.1 kcal/mol), demonstrate decreased affinity towards other examined Abs. The same is true for their affinity to ACE2 which is dropped by 2.7–6.9 kcal/mol. Furthermore, a positive correlation between the RBD-Ab ΔΔGs and the RBD-ACE2 ΔΔGs can be noted (Figure 6B, Pearson’s r = 0.63–0.90) similar to the correlation observed for single mutations and discussed in the previous section. It does not exclude, however, the presence of some RBD variants for which the affinity towards specific Abs is decreased by ~2–4 kcal/mol while there is no notable change of affinity to ACE2 (Figure 6B), although for CC12.1 (6XC2) Ab the effect is lower and does not exceed 2 kcal/mol, apparently for the same reason discussed with respect to the single mutations in the previous section, i.e., it is due to the largest overlap of the CC12.1 Ab epitope with the ACE2 interface, making those RBD mutations, which are unfavorable for the binding of this particular Ab, also adverse for the RBD interaction with ACE2. On the other hand, one cannot exclude a possibility that variants escaping even Abs targeting this epitope, which seem the most resistant to the development of resistance, and, at the same time, interacting in the normal way with ACE2 can appear in future given that over 2021 a significant number of SARS-CoV-2 variants with multiple mutations in RBD emerged comparing to 2020 (see Figure S5). The analysis of single mutations performed above indicates that as many as six mutations (P337G, R403P, T415C, N422P, N481P, and G502R) may potentially result in the substantial decrease in the RBD affinity to the CC12.1 Ab by ~5.6 kcal/mol while the affinity of this variant to ACE2 would even increase by ~0.5 kcal/mol. The full list of single mutations, which lower the binding affinities between RBD and Abs (ΔΔG > 0.6 kcal/mol) but do not lower the binding affinity to ACE2 (ΔΔG ≤ 0 kcal/mol), is provided in Table S1.

Overall, the analysis of RBD variants with multiple mutations suggests the emergence of novel SARS-CoV-2 variants with multiple co-occurring mutations as the major risk factor in terms of resistance development against Abs.

## 4. Conclusions

The great effort with which the scientific community is studying SARS-CoV-2 has generated a huge amount of data about this novel coronavirus since its emergence about two years ago. The open databases of sequences and atomic structures provide an opportunity to perform the high-throughput analysis of interactions between various monoclonal neutralizing antibodies (Abs), hACE2, serving as the main entry point for coronavirus, and their principal viral binding partner, the receptor-binding domain (RBD) of Spike. Here, we employed a theoretical approach to predict the effects of the known and conceivable RBD mutations on the binding energy between RBD, ACE2 and a set of selected representative Abs targeting structurally distinct epitopes, and augmented this analysis with the sequence-level data about the existing SARS-CoV-2 variants.

An analysis of epitopes revealed four major clusters of Ab binding sites differing in both of the involved RBD residues, their conservativity, and degree of the glycan shielding. The assessment of epitope conservativity suggested that Abs belonging to one of the identified clusters (including, among others, CR3022) are likely immunosubdominant despite their seemingly high affinity to RBD. Although most mutations that are currently widely spread appear in the least conservative part of RBD, the receptor-binding motif (RBM), we show that there also exist potentially deleterious mutations in more conservative regions of RBD, which can significantly impair its binding to some Abs. While certain point replacements in RBD, including those previously characterized experimentally and/or observed in the population, can reduce the binding affinity for specific Abs or even several types of Abs to a certain degree, the cumulative effects of multiple mutations may be much more hazardous, thereby leading to resistance against a broader spectrum of Abs as demonstrated by the recently emerged omicron variant. By analyzing sequences of the existing SARS-CoV-2 variants, we identified a number of such naturally occurring mutants potentially capable of extensive antigenic escape.

We expect that our theoretical study will promote future experimental work, complement the eventual prognosis of the properties of the novel SARS-CoV-2 variants, and help to design more efficient treatments for COVID-19. Particularly, the knowledge-based selection of antibody mixtures with non-overlapping escape mutations should reduce the emergence of resistance and prolong the utility of antibody therapies.

## Figures and Tables

**Table 1:** Overview of the analyzed antibody-RBD complexes. Complexes, which are the centers of the clusters in Figure 1, are highlighted in bold. Some structures represent complexes with multiple Abs; therefore, for each complex the specific protein chains corresponding to RBD and Ab heavy/light chains are indicated (only one chain is shown for nanobodies).

Cluster	Complex	ΔG, kcal/mol	No. Contacts	Mean Interface Variability, Bit	Antibody Type/Name	PDB Chains			Ref.
						RBD	H	L	
1	6XDG_CA	−10.7	49	0.09	REGN10987 antibody Fab	E	C	A	[25]
1	6ZBP_B	−9.6	53	0.105	H11-H4 nanobody	A	B		-
1	6XKP_HL	−9.3	52	0.092	neutralizing antibody CV07-270	A	H	L	[26]
1	7BWJ_HL	−9.6	56	0.116	P2B-2F6 Fab	E	H	L	[27]
1	6ZCZ_F	−9.6	55	0.097	nanobody H11-H4	E	F		[28]
1	7C8V_A	−9.9	54	0.145	synthetic nanobody SR4	B	A		-
1	7CHC_AB	−9.5	58	0.106	BD-368-2 Fab	R	A	B	[29]
**1**	**7K9Z_HL**	**−11.5**	**75**	**0.081**	**Fab fragment neutralizing antibody 52**	**E**	**H**	**L**	[30]
1	7JX3_AB	−9.8	63	0.064	Fab domain of monoclonal antibody S309	R	A	B	[31]
2	7JX3_CD	−11.2	82	0.09	Fab domain of monoclonal antibody S2H14	R	C	D	[31]
2	7C8W_A	−11.2	79	0.091	synthetic nanobody MR17	B	A		-
2	6XDG_BD	−10.2	69	0.102	REGN10933 antibody Fab	E	B	D	[25]
2	6XKQ_HL	−9.8	70	0.115	neutralizing antibody CV07-250	A	H	L	[26]
2	7JV2_HL	−10.3	56	0.094	S2H13 neutralizing antibody Fab fragment	A	H	L	[31]
2	7K45_HL	−10.9	66	0.1	S2E12 neutralizing antibody Fab	B	H	L	[32]
2	7K9Z_AB	−9.7	56	0.132	Fab fragment neutralizing antibody 298	E	A	B	[30]
**2**	**7CAN_A**	**−11.5**	**77**	**0.088**	**synthetic nanobody MR17-K99Y**	**B**	**A**		-
2	7JMP_HL	−8.3	52	0.101	neutralizing antibody COVA2-39	A	H	L	[33]
3	6ZCZ_HL	−12.3	80	0.038	EY6A Fab	E	H	L	[28]
3	7CAH_ED	−12.6	86	0.045	H014 Fab	A	E	D	[34]
3	7JMW_HL	−10.7	58	0.039	cross-neutralizing antibody COVA1-16 Fab	A	H	L	[35]
**3**	**6YLA_HL**	**−14.9**	**99**	**0.04**	**CR3022 Fab**	**E**	**H**	**L**	[36]
3	7JX3_HL	−12.4	83	0.042	Fab domain of monoclonal antibody S304	R	H	L	[31]
3	7JVA_HL	−10.2	72	0.049	S2A4 neutralizing antibody Fab fragment	A	H	L	[31]
3	7A5S_HL	−14	102	0.047	CR3022 Fab	A	H	L	[37]
4	7C01_HL	−13.4	112	0.094	neutralizing antibody CB6	A	H	L	[38]
4	7CH5_HL	−12.5	97	0.101	BD-629 Fab	R	H	L	[29]
**4**	**6XC2_HL**	**−16.4**	**137**	**0.083**	**neutralizing antibody CC12.1**	**A**	**H**	**L**	[39]
4	7CH4_HL	−15.9	114	0.09	BD-604 Fab	R	H	L	[29]
4	7BZ5_HL	−14.6	121	0.088	neutralizing antibody B38	A	H	L	[40]
4	7JMO_HL	−12.9	108	0.086	neutralizing antibody COVA2-04	A	H	L	[33]
4	7CHB_HL	−13.5	110	0.087	BD-236 Fab	R	H	L	[29]
4	6XC4_HL	−14	99	0.104	neutralizing antibody CC12.3	A	H	L	[39]
4	7CHC_HL	−12	95	0.101	BD-629 Fab	R	H	L	[29]
4	7K8M_AB	−13.5	110	0.093	Fab fragment neutralizing antibody C102	E	A	B	[41]
-	6M17_EB	−11.4	66	0.101	ACE2 receptor	E	B		[24]

**Table 2:** SARS-CoV-2 variants of concern and variants of interest considered by WHO along with the corresponding mutations in RBD.

Variant(s)	RBD Mutations
B.1.1.7 (*α*)	N501Y
B.1.351 (*β*)	K417N, E484K, N501Y
P.1 (*γ*)	K417T, E484K, N501Y
B.1.617.2 (*δ*)	L452R, T478K
B.1.617.2+ (*δ+*)	L452R, K417N, T478K
B.1.427/B.1.429 (*ε*)	L452R
P.2, B.1.525, B.1.526 (*ζ*, *η*, *ι*)	E484K
P.3 (*θ*)	E484K, N501Y
B.1.617.1 (*κ*)	L452R, E484Q
C.37 (*λ*)	L452Q, F490S
B.1.621 (*μ*)	R346K, E484K, N501Y
B.1.1.529 (*ο*)	G339D, S371L, S373P, S375F, K417N, N440K, G446S, S477N, T478K, E484A, Q493R, G496S, Q498R, N501Y, Y505H

**Table 3:** Predicted change of the RBD-Ab/ACE2 binding energy for some existing SARS-CoV2 variants with multiple mutations.

ID	Origin	No. Mutations	ΔΔG, kcal/mol				
			RBD:7K9Z	RBD:7CAN	RBD:6YLA	RBD:6XC2	RBD:ACE2
EPI_ISL_410541	pangolin/Guangxi	25	4.68	9.52	3.41	8.21	6.93
EPI_ISL_410538	pangolin/Guangxi	24	3.78	8.46	2.77	7.54	5.81
EPI_ISL_410539	pangolin/Guangxi	23	3.21	8.2	2.54	7.41	5.58
EPI_ISL_402131	bat/Yunnan	20	3.46	7.37	2.08	7.78	5.6
EPI_ISL_568499	human/Iran	8	7.27	5.02	4.83	5.31	4.44
EPI_ISL_568500	human/Iran	11	3.9	3.35	4.37	2.65	2.71
B.1.351 (β)		3	0.3	0.48	0.13	0.8	0.29
B.1.617.2+ (δ+)		3	1.49	0.87	0.55	1.39	0.58
B.1.621 (μ)		3	0.5	0.21	−0.19	−0.23	−0.16
B.1.1.529 (ο)		15	1.44	4.2	1.08	5.59	4.92

## Supplementary Materials

The following supporting information can be downloaded at: https://www.mdpi.com/article/10.3390/v14020295/s1, Figure S1: Theoretical estimation of the free energy of the Ab-RBD complexes plotted as a function of the contact count. The linear regression trend is shown.; Figure S2: Number of novel unique mutations appeared at the RBD interfaces of selected Abs and ACE2 during the course of pandemics; Figure S3: Shannon entropy and per-residue change of solvent accessible surface area (SASA) for RBD residues; Figure S4: Mutual Information (MI) matrix estimated from the MSA of RBD; Figure S5: Number of RBD sequences stored in the GISAID database during 2020 and during 2020-2021 periods with the given mutation counts with respect to the original Wuhan variant. Number of multiple mutants increases significantly in 2021 compared to 2020; Table S1: List of mutations, which lower the binding affinities between RBD and antibodies (ΔΔG > 0.6 kcal/mol) but do not lower the binding affinity to ACE2 (ΔΔG ≤ 0 kcal/mol).

## References

[1] Krammer F. (2020). SARS-CoV-2 Vaccines in Development. Nature.

[2] Xiaojie S., Yu L., Lei Y., Guang Y., Min Q. (2020). Neutralizing Antibodies Targeting SARS-CoV-2 Spike Protein. Stem Cell Res..

[3] Li W., Moore M.J., Vasilieva N., Sui J., Wong S.K., Berne M.A., Somasundaran M., Sullivan J.L., Luzuriaga K., Greenough T.C. (2003). Angiotensin-Converting Enzyme 2 Is a Functional Receptor for the SARS Coronavirus. Nature.

[4] Shang J., Ye G., Shi K., Wan Y., Luo C., Aihara H., Geng Q., Auerbach A., Li F. (2020). Structural Basis of Receptor Recognition by SARS-CoV-2. Nature.

[5] Jackson C.B., Farzan M., Chen B., Choe H. (2022). Mechanisms of SARS-CoV-2 Entry into Cells. Nat. Rev. Mol. Cell Biol..

[6] Chi X., Yan R., Zhang J., Zhang G., Zhang Y., Hao M., Zhang Z., Fan P., Dong Y., Yang Y. (2020). A Neutralizing Human Antibody Binds to the N-Terminal Domain of the Spike Protein of SARS-CoV-2. Science.

[7] Tong P., Gautam A., Windsor I.W., Travers M., Chen Y., Garcia N., Whiteman N.B., McKay L.G.A., Storm N., Malsick L.E. (2021). Memory B Cell Repertoire for Recognition of Evolving SARS-CoV-2 Spike. Cell.

[8] Gaebler C., Wang Z., Lorenzi J.C.C., Muecksch F., Finkin S., Tokuyama M., Cho A., Jankovic M., Schaefer-Babajew D., Oliveira T.Y. (2021). Evolution of Antibody Immunity to SARS-CoV-2. Nature.

[9] Harvey W.T., Carabelli A.M., Jackson B., Gupta R.K., Thomson E.C., Harrison E.M., Ludden C., Reeve R., Rambaut A., COVID-19 Genomics UK (COG-UK) Consortium (2021). SARS-CoV-2 Variants, Spike Mutations and Immune Escape. Nat. Rev. Microbiol..

[10] Alam I., Radovanovic A., Incitti R., Kamau A.A., Alarawi M., Azhar E.I., Gojobori T. (2021). CovMT: An Interactive SARS-CoV-2 Mutation Tracker, with a Focus on Critical Variants. Lancet Infect. Dis..

[11] Wang P., Nair M.S., Liu L., Iketani S., Luo Y., Guo Y., Wang M., Yu J., Zhang B., Kwong P.D. (2021). Antibody Resistance of SARS-CoV-2 Variants B.1.351 and B.1.1.7. Nature.

[12] Dawood A.A. (2020). Mutated COVID. New Microbes New Infect..

[13] Cui J., Li F., Shi Z.-L. (2019). Origin and Evolution of Pathogenic Coronaviruses. Nat. Rev. Microbiol..

[14] Weisblum Y., Schmidt F., Zhang F., DaSilva J., Poston D., Lorenzi J.C., Muecksch F., Rutkowska M., Hoffmann H.-H., Michailidis E. (2020). Escape from Neutralizing Antibodies by SARS-CoV-2 Spike Protein Variants. eLife.

[15] Satyam R., Yousef M., Qazi S., Bhat A.M., Raza K. (2021). COVIDium: A COVID-19 Resource Compendium. Database.

[16] Ahsan M.A., Liu Y., Feng C., Hofestädt R., Chen M. (2021). OverCOVID: An Integrative Web Portal for SARS-CoV-2 Bioinformatics Resources. J. Integr. Bioinform..

[17] Shu Y., McCauley J. (2017). GISAID: Global Initiative on Sharing All Influenza Data—From Vision to Reality. Euro Surveill..

[18] Ahsan M.A., Liu Y., Feng C., Zhou Y., Ma G., Bai Y., Chen M. (2021). Bioinformatics Resources Facilitate Understanding and Harnessing Clinical Research of SARS-CoV-2. Brief. Bioinform..

[19] Starr T.N., Greaney A.J., Addetia A., Hannon W.W., Choudhary M.C., Dingens A.S., Li J.Z., Bloom J.D. (2021). Prospective Mapping of Viral Mutations That Escape Antibodies Used to Treat COVID-19. Science.

[20] Thomson E.C., Rosen L.E., Shepherd J.G., Spreafico R., da Silva Filipe A., Wojcechowskyj J.A., Davis C., Piccoli L., Pascall D.J., Dillen J. (2021). Circulating SARS-CoV-2 Spike N439K Variants Maintain Fitness While Evading Antibody-Mediated Immunity. Cell.

[21] Li Q., Nie J., Wu J., Zhang L., Ding R., Wang H., Zhang Y., Li T., Liu S., Zhang M. (2021). No Higher Infectivity but Immune Escape of SARS-CoV-2 501Y.V2 Variants. Cell.

[22] Wrapp D., Wang N., Corbett K.S., Goldsmith J.A., Hsieh C.-L., Abiona O., Graham B.S., McLellan J.S. (2020). Cryo-EM Structure of the 2019-NCoV Spike in the Prefusion Conformation. Science.

[23] Walls A.C., Park Y.-J., Tortorici M.A., Wall A., McGuire A.T., Veesler D. (2020). Structure, Function, and Antigenicity of the SARS-CoV-2 Spike Glycoprotein. Cell.

[24] Yan R., Zhang Y., Li Y., Xia L., Guo Y., Zhou Q. (2020). Structural Basis for the Recognition of SARS-CoV-2 by Full-Length Human ACE2. Science.

[25] Hansen J., Baum A., Pascal K.E., Russo V., Giordano S., Wloga E., Fulton B.O., Yan Y., Koon K., Patel K. (2020). Studies in Humanized Mice and Convalescent Humans Yield a SARS-CoV-2 Antibody Cocktail. Science.

[26] Kreye J., Reincke S.M., Kornau H.-C., Sánchez-Sendin E., Corman V.M., Liu H., Yuan M., Wu N.C., Zhu X., Lee C.-C.D. (2020). A Therapeutic Non-Self-Reactive SARS-CoV-2 Antibody Protects from Lung Pathology in a COVID-19 Hamster Model. Cell.

[27] Ju B., Zhang Q., Ge J., Wang R., Sun J., Ge X., Yu J., Shan S., Zhou B., Song S. (2020). Human Neutralizing Antibodies Elicited by SARS-CoV-2 Infection. Nature.

[28] Zhou D., Duyvesteyn H.M.E., Chen C.-P., Huang C.-G., Chen T.-H., Shih S.-R., Lin Y.-C., Cheng C.-Y., Cheng S.-H., Huang Y.-C. (2020). Structural Basis for the Neutralization of SARS-CoV-2 by an Antibody from a Convalescent Patient. Nat. Struct. Mol. Biol..

[29] Du S., Cao Y., Zhu Q., Yu P., Qi F., Wang G., Du X., Bao L., Deng W., Zhu H. (2020). Structurally Resolved SARS-CoV-2 Antibody Shows High Efficacy in Severely Infected Hamsters and Provides a Potent Cocktail Pairing Strategy. Cell.

[30] Rujas E., Kucharska I., Tan Y.Z., Benlekbir S., Cui H., Zhao T., Wasney G.A., Budylowski P., Guvenc F., Newton J.C. (2021). Multivalency transforms SARS-CoV-2 antibodies into ultrapotent neutralizers. Nat. Commun..

[31] Piccoli L., Park Y.-J., Tortorici M.A., Czudnochowski N., Walls A.C., Beltramello M., Silacci-Fregni C., Pinto D., Rosen L.E., Bowen J.E. (2020). Mapping Neutralizing and Immunodominant Sites on the SARS-CoV-2 Spike Receptor-Binding Domain by Structure-Guided High-Resolution Serology. Cell.

[32] Tortorici M.A., Beltramello M., Lempp F.A., Pinto D., Dang H.V., Rosen L.E., McCallum M., Bowen J., Minola A., Jaconi S. (2020). Ultrapotent Human Antibodies Protect against SARS-CoV-2 Challenge via Multiple Mechanisms. Science.

[33] Wu N.C., Yuan M., Liu H., Lee C.-C.D., Zhu X., Bangaru S., Torres J.L., Caniels T.G., Brouwer P.J.M., van Gils M.J. (2020). An Alternative Binding Mode of IGHV3-53 Antibodies to the SARS-CoV-2 Receptor Binding Domain. Cell Rep..

[34] Lv Z., Deng Y.-Q., Ye Q., Cao L., Sun C.-Y., Fan C., Huang W., Sun S., Sun Y., Zhu L. (2020). Structural Basis for Neutralization of SARS-CoV-2 and SARS-CoV by a Potent Therapeutic Antibody. Science.

[35] Liu H., Wu N.C., Yuan M., Bangaru S., Torres J.L., Caniels T.G., van Schooten J., Zhu X., Lee C.-C.D., Brouwer P.J.M. (2020). Cross-Neutralization of a SARS-CoV-2 Antibody to a Functionally Conserved Site Is Mediated by Avidity. bioRxiv.

[36] Huo J., Zhao Y., Ren J., Zhou D., Duyvesteyn H.M.E., Ginn H.M., Carrique L., Malinauskas T., Ruza R.R., Shah P.N.M. (2020). Neutralization of SARS-CoV-2 by Destruction of the Prefusion Spike. Cell Host Microbe.

[37] Wrobel A.G., Benton D.J., Hussain S., Harvey R., Martin S.R., Roustan C., Rosenthal P.B., Skehel J.J., Gamblin S.J. (2020). Antibody-Mediated Disruption of the SARS-CoV-2 Spike Glycoprotein. Nat. Commun..

[38] Shi R., Shan C., Duan X., Chen Z., Liu P., Song J., Song T., Bi X., Han C., Wu L. (2020). A Human Neutralizing Antibody Targets the Receptor-Binding Site of SARS-CoV-2. Nature.

[39] Yuan M., Liu H., Wu N.C., Lee C.-C.D., Zhu X., Zhao F., Huang D., Yu W., Hua Y., Tien H. (2020). Structural Basis of a Shared Antibody Response to SARS-CoV-2. Science.

[40] Wu Y., Wang F., Shen C., Peng W., Li D., Zhao C., Li Z., Li S., Bi Y., Yang Y. (2020). A Noncompeting Pair of Human Neutralizing Antibodies Block COVID-19 Virus Binding to Its Receptor ACE2. Science.

[41] Barnes C.O., Jette C.A., Abernathy M.E., Dam K.-M.A., Esswein S.R., Gristick H.B., Malyutin A.G., Sharaf N.G., Huey-Tubman K.E., Lee Y.E. (2020). SARS-CoV-2 Neutralizing Antibody Structures Inform Therapeutic Strategies. Nature.

[42] Vangone A., Bonvin A.M. (2015). Contacts-Based Prediction of Binding Affinity in Protein-Protein Complexes. Elife.

[43] Xue L.C., Rodrigues J.P., Kastritis P.L., Bonvin A.M., Vangone A. (2016). PRODIGY: A Web Server for Predicting the Binding Affinity of Protein-Protein Complexes. Bioinformatics.

[44] Dehouck Y., Kwasigroch J.M., Rooman M., Gilis D. (2013). BeAtMuSiC: Prediction of Changes in Protein-Protein Binding Affinity on Mutations. Nucleic Acids Res..

[45] Geng C., Xue L.C., Roel-Touris J., Bonvin A.M.J.J. (2019). Finding the ΔΔ G Spot: Are Predictors of Binding Affinity Changes upon Mutations in Protein–Protein Interactions Ready for It?. Wiley Interdiscip. Rev. Comput. Mol. Sci..

[46] GISAID—Initiative. https://www.gisaid.org/.

[47] Katoh K., Standley D.M. (2013). MAFFT Multiple Sequence Alignment Software Version 7: Improvements in Performance and Usability. Mol. Biol. Evol..

[48] Bakan A., Dutta A., Mao W., Liu Y., Chennubhotla C., Lezon T.R., Bahar I. (2014). Evol and ProDy for Bridging Protein Sequence Evolution and Structural Dynamics. Bioinformatics.

[49] Dunn S.D., Wahl L.M., Gloor G.B. (2008). Mutual Information without the Influence of Phylogeny or Entropy Dramatically Improves Residue Contact Prediction. Bioinformatics.

[50] Woo H., Park S.-J., Choi Y.K., Park T., Tanveer M., Cao Y., Kern N.R., Lee J., Yeom M.S., Croll T.I. (2020). Developing a Fully Glycosylated Full-Length SARS-CoV-2 Spike Protein Model in a Viral Membrane. J. Phys. Chem. B.

[51] CHARMM-GUI. https://charmm-gui.org/?doc=archive&lib=covid19.

[52] Abraham M.J., Murtola T., Schulz R., Páll S., Smith J.C., Hess B., Lindahl E. (2015). GROMACS: High Performance Molecular Simulations through Multi-Level Parallelism from Laptops to Supercomputers. SoftwareX.

[53] Tortorici M.A., Veesler D. (2019). Structural Insights into Coronavirus Entry. Adv. Virus Res..

[54] Grant O.C., Montgomery D., Ito K., Woods R.J. (2020). Analysis of the SARS-CoV-2 Spike Protein Glycan Shield Reveals Implications for Immune Recognition. Sci. Rep..

[55] Barton M.I., MacGowan S.A., Kutuzov M.A., Dushek O., Barton G.J., van der Merwe P.A. (2021). Effects of Common Mutations in the SARS-CoV-2 Spike RBD and Its Ligand, the Human ACE2 Receptor on Binding Affinity and Kinetics. eLife.

[56] Yuan M., Wu N.C., Zhu X., Lee C.-C.D., So R.T.Y., Lv H., Mok C.K.P., Wilson I.A. (2020). A Highly Conserved Cryptic Epitope in the Receptor Binding Domains of SARS-CoV-2 and SARS-CoV. Science.

[57] Phillips P.C. (2008). Epistasis--the Essential Role of Gene Interactions in the Structure and Evolution of Genetic Systems. Nat. Rev. Genet..

[58] Greaney A.J., Starr T.N., Barnes C.O., Weisblum Y., Schmidt F., Caskey M., Gaebler C., Cho A., Agudelo M., Finkin S. (2021). Mapping Mutations to the SARS-CoV-2 RBD That Escape Binding by Different Classes of Antibodies. Nat. Commun..

[59] Morcos F., Onuchic J.N. (2019). The Role of Coevolutionary Signatures in Protein Interaction Dynamics, Complex Inference, Molecular Recognition, and Mutational Landscapes. Curr. Opin. Struct. Biol..

[60] Wu N.C., Xie J., Zheng T., Nycholat C.M., Grande G., Paulson J.C., Lerner R.A., Wilson I.A. (2017). Diversity of Functionally Permissive Sequences in the Receptor-Binding Site of Influenza Hemagglutinin. Cell Host Microbe.

[61] Li Q., Wu J., Nie J., Zhang L., Hao H., Liu S., Zhao C., Zhang Q., Liu H., Nie L. (2020). The Impact of Mutations in SARS-CoV-2 Spike on Viral Infectivity and Antigenicity. Cell.

[62] Yi C., Sun X., Ye J., Ding L., Liu M., Yang Z., Lu X., Zhang Y., Ma L., Gu W. (2020). Key Residues of the Receptor Binding Motif in the Spike Protein of SARS-CoV-2 That Interact with ACE2 and Neutralizing Antibodies. Cell. Mol. Immunol..

[63] Ku Z., Xie X., Davidson E., Ye X., Su H., Menachery V.D., Li Y., Yuan Z., Zhang X., Muruato A.E. (2021). Molecular Determinants and Mechanism for Antibody Cocktail Preventing SARS-CoV-2 Escape. Nat. Commun..

[64] Callaway E. (2021). Could New COVID Variants Undermine Vaccines? Labs Scramble to Find Out. Nature.

[65] Abdelrahman Z., Li M., Wang X. (2020). Comparative Review of SARS-CoV-2, SARS-CoV, MERS-CoV, and Influenza A Respiratory Viruses. Front. Immunol..

[66] Tian X., Li C., Huang A., Xia S., Lu S., Shi Z., Lu L., Jiang S., Yang Z., Wu Y. (2020). Potent Binding of 2019 Novel Coronavirus Spike Protein by a SARS Coronavirus-Specific Human Monoclonal Antibody. Emerg. Microbes Infect..

[67] Planas D., Saunders N., Maes P., Guivel-Benhassine F., Planchais C., Buchrieser J., Bolland W.-H., Porrot F., Staropoli I., Lemoine F. (2021). Considerable Escape of SARS-CoV-2 Omicron to Antibody Neutralization. Nature.

[68] Wolter N., Jassat W., Walaza S., Welch R., Moultrie H., Groome M., Amoako D.G., Everatt J., Bhiman J.N., Scheepers C. (2021). Early Assessment of the Clinical Severity of the SARS-CoV-2 Omicron Variant in South Africa. bioRxiv.

