# Supplementary material for: Computational Analysis of Mutations in the Receptor-Binding Domain of SARS-CoV-2 Spike and Their Effects on Antibody Binding
Source: Viruses. 2022 Jan 30;14(2):295. doi: 10.3390/v14020295 (PMC8874930; doi:10.3390/v14020295)
Supplement: Supplementary file 1 [file viruses-14-00295-s001.zip › viruses-1552276-supplementary.pdf]

Computational Analysis of Mutations in the Receptor-Binding Domain of SARS-CoV-2 Spike and their Effects on Antibody Binding

Marine E. Bozdaganyan[a,b,c], Konstantin V. Shaitan[a,b], Mikhail P. Kirpichnikov[a], Olga S. Sokolova[a,c]*, and Philipp S. Orekhov[a,c,d]*

[a] Faculty of Biology, M.V. Lomonosov Moscow State University, Moscow, Russia
[b] N.N. Semenov Federal Research Center for Chemical Physics, Russian Academy of Sciences, Moscow, Russia
[c] Shenzhen MSU-BIT University, Shenzhen, China
[d] Sechenov University, Moscow, Russia

* corresponding authors, e-mail: sokolova@mail.bio.msu.ru; orekhov@mail.bio.msu.ru

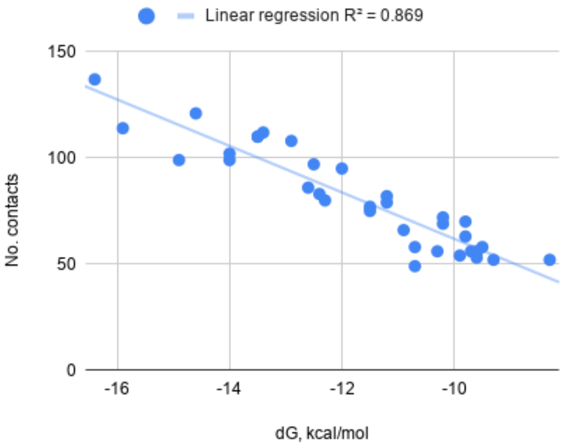

Figure S1. Theoretical estimation of the free energy of the Ab-RBD complexes plotted as a function of the contact count. The linear regression trend is shown.

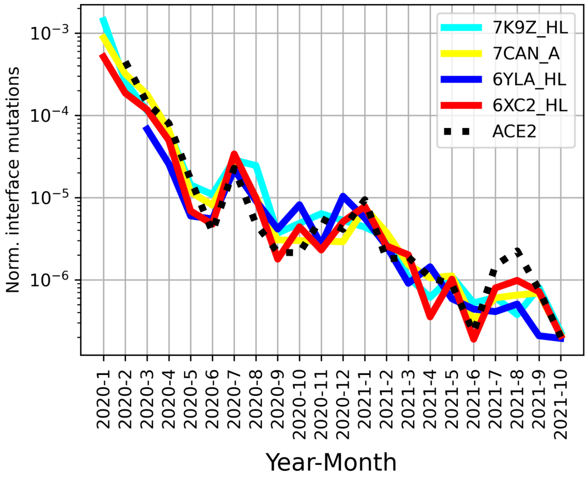

Figure S2. Number of novel unique mutations appeared at the RBD interfaces for 4 selected Abs (color lines) and ACE2 (dashed black line) normalized by the epitope size and the number of RBD sequences during the course of pandemics (from March 2020 to October 2021).

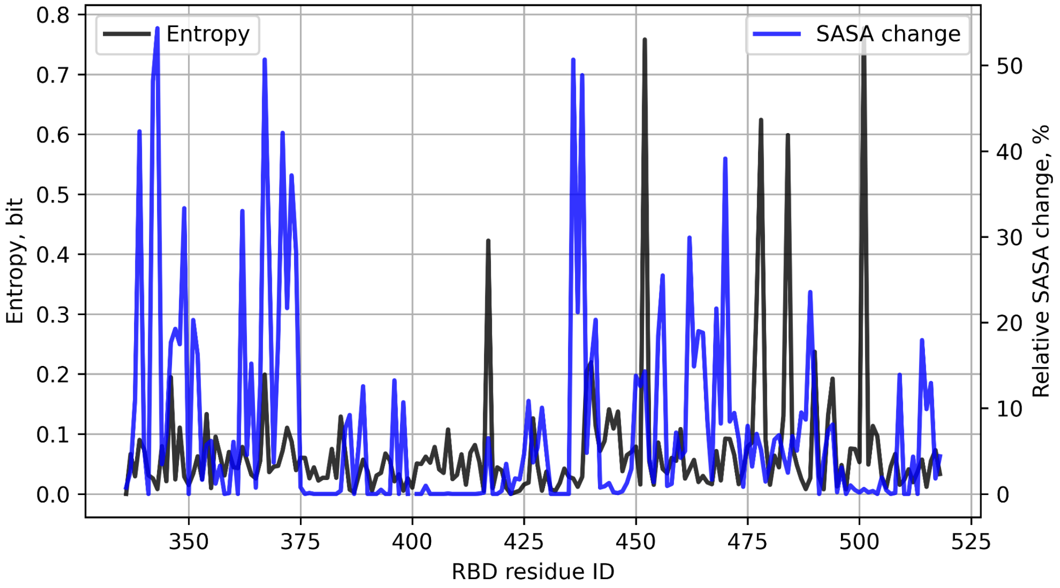

Figure S3. Shannon entropy calculated using the multiple sequence alignment (MSA) of RBD (black curve) and per-residue change of solvent accessible surface area (SASA) in the presence of glycans with respect to the unglycosylated S-protein (blue curve).

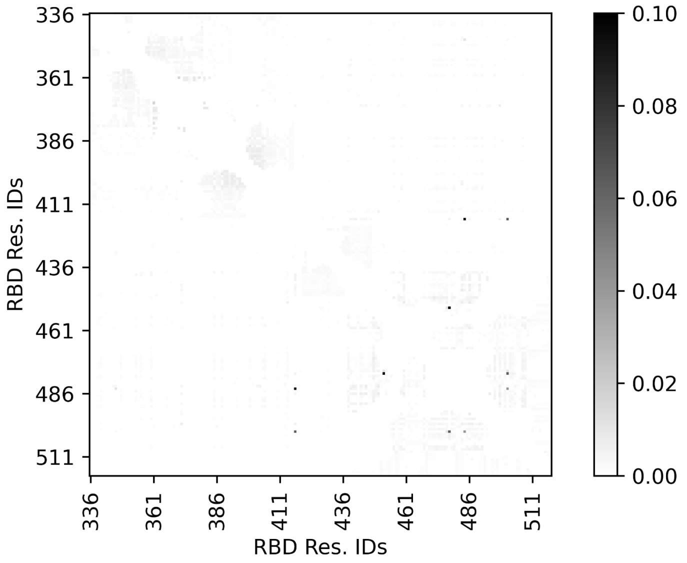

Figure S4. Mutual Information (MI) matrix estimated from the MSA of RBD indicating several spatially distant co-evolving positions. Mutations in variable positions can occur because they are either accompanied or preceded by compensatory changes in other variable positions. Such compensation would result in a coupling between changes in the two positions, or coevolution. The degree of such coupling can be estimated by means of mutual information (MI). Top 5

co-evolving pairs of positions are N501-E484, N501-T478, N501-K417, E484-K417, and T478-L452.

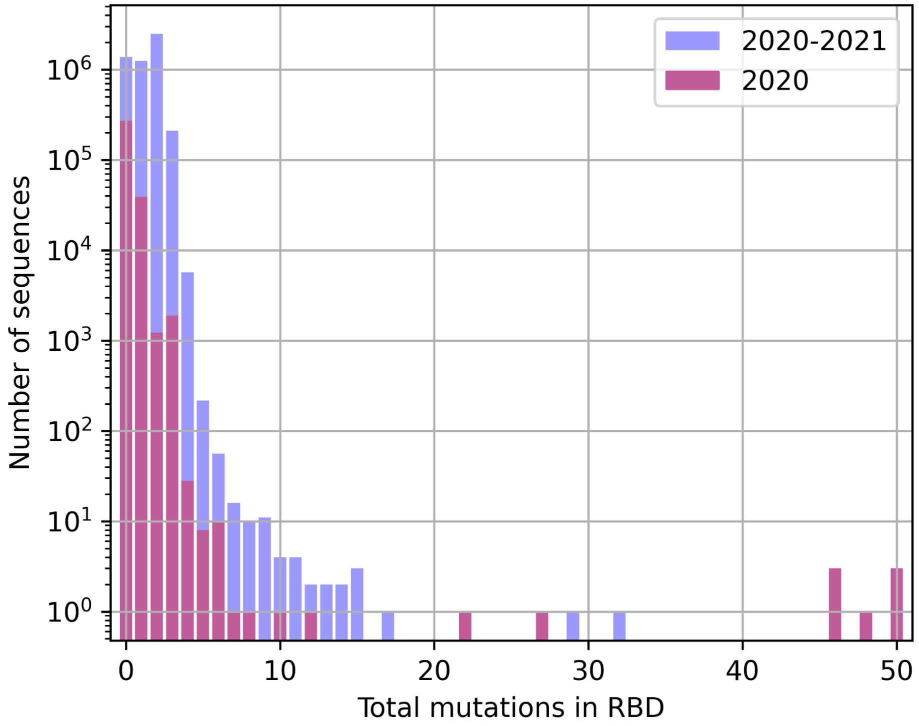

Figure S5. Number of RBD sequences stored in the GISAID database during 2020 and during 2020-2021 periods with the given mutation counts with respect to the original Wuhan variant. Number of multiple mutants increases significantly in 2021 compared to 2020.

Table S1. List of mutations, which lower the binding affinities between RBD and antibodies ($\Delta\Delta G > 0.6$ kcal/mol) but do not lower the binding affinity to ACE2 ($\Delta\Delta G \leq 0$ kcal/mol).

Antibody	Variant	ΔΔG, kcal/mol (RBD:Ab)	ΔΔG, kcal/mol (RBD:ACE2)
7K9Z_HL	P337M	0.62	-0.22
7K9Z_HL	P337R	0.62	-0.26
7K9Z_HL	P337E	0.63	-0.16
7K9Z_HL	P337Q	0.63	-0.19
7K9Z_HL	P337G	0.64	-0.06
7K9Z_HL	V483C	0.66	-0.01
7K9Z_HL	P337Y	0.76	-0.26
7K9Z_HL	P337F	0.77	-0.24
7K9Z_HL	P337W	0.81	-0.15
7K9Z_HL	I468F	0.81	0
7K9Z_HL	I468H	0.88	-0.02
7K9Z_HL	G482C	0.89	0

7K9Z_HL	V483G	1.02	-0.34
7K9Z_HL	I468T	1.77	-0.08
7K9Z_HL	I468N	1.83	-0.01
7CAN_A	E484S	0.61	0
7CAN_A	S383P	0.65	-0.05
7CAN_A	P337W	0.67	-0.15
7CAN_A	V483G	0.82	-0.34
7CAN_A	E484A	0.83	-0.01
6YLA_HL	V362A	0.64	0
6YLA_HL	D428T	0.66	-0.04
6YLA_HL	P337K	0.68	-0.18
6YLA_HL	P337E	0.7	-0.16
6YLA_HL	P337G	0.72	-0.06
6YLA_HL	Y369L	0.72	-0.1
6YLA_HL	K378T	0.76	-0.19
6YLA_HL	T385N	0.85	-0.13
6YLA_HL	K378Q	0.88	0
6YLA_HL	K378C	0.93	-0.12
6YLA_HL	D428A	0.95	0
6YLA_HL	K378S	1.21	-0.07
6YLA_HL	T385E	1.23	-0.09
6YLA_HL	K378N	1.24	-0.05
6YLA_HL	T385D	1.24	-0.17
6YLA_HL	T385Q	1.24	-0.02
6YLA_HL	G381A	1.25	-0.04
6YLA_HL	G381S	1.26	-0.07
6YLA_HL	Y369I	1.33	-0.02
6YLA_HL	S383P	1.35	-0.05
6YLA_HL	T385S	1.36	-0.09
6YLA_HL	T385P	1.5	-0.05
6XC2_HL	N481P	0.64	-0.11
6XC2_HL	R403L	0.66	-0.04
6XC2_HL	P337K	0.67	-0.18
6XC2_HL	R403I	0.67	-0.01
6XC2_HL	P337E	0.68	-0.16
6XC2_HL	N422P	0.68	-0.18
6XC2_HL	T415R	0.75	-0.03

6XC2_HL	P337G	0.84	-0.06
6XC2_HL	T415C	1.01	-0.02
6XC2_HL	G502R	1.07	-0.01
6XC2_HL	R403P	1.36	-0.05
